# Validity of clinical associations of biomarkers in translational research studies: the case of systemic autoimmune diseases

**DOI:** 10.1186/ar3143

**Published:** 2010-09-27

**Authors:** Maria G Tektonidou, Michael M Ward

**Affiliations:** 1 Intramural Research Program, National Institute of Arthritis and Musculoskeletal and Skin Diseases, National Institutes of Health, 10 Center Drive, Bethesda, MD 20892, USA; 2 Euroclinic Hospital, 9 Athanassiadou Street, 11521 Athens, and First Department of Internal Medicine, School of Medicine, National University of Athens, 75 M Asias Street, 11527 Athens, Greece

## Abstract

**Introduction:**

Validity of biomarkers may be affected if studies do not include certain features in their design. We evaluated whether translational research studies of potential biomarkers incorporated design features important for valid clinical associations.

**Methods:**

We searched 10 journals for translational studies in six systemic autoimmune diseases published in 2004 through 2009. We included studies that reported associations between laboratory markers and the presence of disease, measures of disease activity, or prognosis. We examined the following design features: age, sex, and race matching; control for effects of treatment on expression of the biomarker; inclusion of patients with both early and late disease, or both active and inactive disease; longitudinal or cross-sectional design; and use of validated activity and damage measures.

**Results:**

Among 170 articles, 156 articles examined potential biomarkers for diagnosis, 37 for disease activity assessment, and nine for prognosis; 67 were studies of rheumatoid arthritis (RA); 48, of systemic lupus erythematosus; and 41, of other diseases. Gene-expression profiles were the most commonly examined potential biomarkers (*n *= 51). Fewer than one half of studies incorporated study-design features important for valid clinical associations. Only 47.4% of studies of biomarkers for diagnosis had groups that were age-matched, 45.5% were sex-matched, and 35.3% controlled for treatment. Studies that examined biomarkers in histologic samples and studies of RA were less likely to include important design features.

**Conclusions:**

Fewer than one half of translational studies of potential biomarkers incorporated design features needed for valid interpretation of clinical associations. Attention to these features could reduce false-positive and false-negative associations.

## Introduction

Dramatic recent progress in immunology has resulted in the recognition of new pathways mediating inflammation and tissue damage in systemic autoimmune diseases. These discoveries have led to the identification of biochemical, molecular, or genetic markers that indicate normal and pathogenic biologic processes or pharmacologic responses [1]. One goal of translational research is to test whether these markers, characterized as biomarkers, have clinical application in improving diagnostic accuracy or assessing disease activity, prognosis, and treatment efficacy [2,3].

In early biomarker testing, the influence of confounding factors and other sources of information bias should be addressed [4-7]. Laboratory scientists are often the first to examine clinical applications of their discoveries. Laboratory scientists may not always design early translational studies with features that guard against confounding and information bias but may rather test samples of convenience. To know whether a biomarker is associated with a disease, matching patients and healthy controls on age and sex would be important.

Medications can also influence biomarker expression and cause comparisons between patients and controls to be spurious [8-10]. Biomarkers can be specific to particular stages of disease (early or late) or particular states of disease activity. False-positive or false-negative associations may occur if studies include only certain subsets of patients. It is not known how often these issues, which can lead to misdirected efforts or premature abandonment of promising lines of research, are considered in early studies of potential biomarkers.

The purpose of our study was to evaluate whether recently published translational research studies that examined biomarkers for diagnosis, disease activity, and prognosis in systemic autoimmune diseases incorporated study-design features important for valid clinical associations.

## Materials and methods

### Data sources and search

We systematically searched the last 5 years (May 2004 through June 2009) of nine basic research journals that potentially included translational research articles on new biomarkers for systemic autoimmune diseases: *Journal of Immunology*, *Journal of Experimental Medicine*, *Journal of Clinical Investigation*, *Nature*, *Nature Medicine*, *Nature Immunology*, *Science*, *Proceedings of the National Academy of Sciences of the USA*, and *Immunity*. We selected these journals because they may include the initial studies of promising biomarkers. Initial studies are informative because they are not influenced by previous findings, but they may influence subsequent development and testing of the biomarker. In addition, among journals that focus on rheumatic diseases, we included *Arthritis and Rheumatism*, the largest rheumatology subspecialty journal, as a representative clinical and basic-research journal. The journals and number of years included were considered to provide a broad survey of recent research.

We searched MEDLINE for the following systemic autoimmune diseases: systemic lupus erythematosus (SLE), rheumatoid arthritis (RA), juvenile idiopathic arthritis (or juvenile rheumatoid arthritis), Sjögren syndrome, anti-neutrophil cytoplasmic antibody-associated vasculitis, and inflammatory myopathies. We included several different diseases to increase the generalizability of the results. We used as key words each disease name in combination with the following terms for categories of potential biomarkers: "cytokines," "cytokine receptors," "chemokines," "chemokine receptors," "antibodies," "enzymes," "endothelial activation markers," "adhesion molecules," "soluble cell-surface molecules," "cell surface markers," "lymphocyte markers," "signaling molecules," "urinary markers," "gene expression," "transcriptomes," and "proteomes." For example, we searched the key-word combination "rheumatoid arthritis" and "gene expression." Separate searches were performed for each combination of disease-name key word and biomarker-category key word.

### Study selection

We included studies that reported associations between a laboratory marker (excluding imaging markers and tests currently used in clinical practice) and either the presence of disease, measures of disease activity, or prognosis. We considered a study to be related *to diagnosis *if it reported the comparison of the potential biomarker between patients with autoimmune disease and healthy controls. For studies examining potential biomarkers for RA diagnosis, we also accepted studies that used patients with osteoarthritis as the control group, as this was a common practice among these studies. We considered a study to be related to *assessment of disease activity *if it reported comparisons of the potential biomarker between patients with active and inactive disease or correlations with measures of disease activity. We considered a study to be related *to prognosis *if it reported associations with organ damage or mortality.

We excluded studies for the following reasons:

1. studies that did not include data on humans (that is, exclusively animal models);

2. studies of genetic polymorphisms, because these studies examine likelihood of disease susceptibility rather than diagnosis;

3. studies of pathogenetic mechanisms and pathways, rather than of specific molecules that could be used as potential biomarkers;

4. studies of treatments or adverse events;

5. studies of other diseases or with a different purpose; and

6. case reports, review articles, or commentaries.

We first screened titles and abstracts of the studies and excluded clearly irrelevant studies. The full text of all remaining studies was read.

### Data extraction

Both authors read each article and abstracted data independently by using a standard data-collection form. Differences were reconciled by discussion. For each study, we recorded the journal, disease examined, number of patients, whether the study included data only on humans or on both humans and animals, category of potential biomarker, and type of specimen (for example, serum, synovial fluid, histologic sample).

We collected data on study-design features important for valid interpretation of clinical associations. These features differed slightly for studies of diagnosis, disease activity, or prognosis assessments (Table 1). If information on a particular feature was not reported, it was considered not to be present.

**Table 1:** Study-design features evaluated in studies examining potential biomarkers

Diagnosis	Disease activity	Prognosis
Matched for age	Matched for age	Matched for age
Matched for sex	Matched for sex	Matched for sex
Matched for race	Matched for race	Matched for race
Provided information on medications	Provided information on medications	Provided information on medications
Controlled for treatment effects	Controlled for treatment effects	Controlled for treatment effects
Reported use of accepted classification criteria	Reported use of validated disease-activity measures	Reported use of validated measures of damage
Included patients with both early and late disease	Longitudinal versus cross-sectional design	Longitudinal versus cross-sectional design
Included patients with both active and inactive disease	Included a wide range of disease-activity scores	
Included disease controls		

#### Studies of biomarkers for diagnosis

We examined nine study-design features for studies that compared patients with unaffected controls. We recorded whether the disease and control groups were individually matched or group matched for age, sex, and race, as these may be important confounding factors. We considered groups to be matched for age if the samples were reported as such by the authors, if the mean or median ages in the disease and control groups were comparable (within 8 years for studies of adults), or if, in the absence of data on mean or median ages, the age ranges of the groups substantially overlapped. We considered studies to be matched for sex and race if they were reported as such or if the proportions in disease and control groups were within 15%. All studies from Asia were considered to be race matched. Methods to adjust for demographic differences other than matching were not used in studies of diagnosis.

Because medications can influence biomarker expression and would likely differ between disease and control groups, we recorded whether information on medications was provided. Additionally, we evaluated whether the authors reported any one of three possible methods to control for confounding by treatment: enrollment of treatment-naïve patients; testing whether biomarker expression differed between groups of patients taking different medications; or providing a reference from the literature regarding associations between the potential biomarker and any medication.

In addition, we recorded whether the study enrolled patients who met accepted classification criteria, to ensure that homogenous groups of patients were studied. We also recorded whether both patients with active disease and patients with inactive disease were enrolled. Inclusion of both groups helps to avoid spectrum bias by ensuring that the biomarker is associated with the disease itself, and not only with a particular level of disease activity [11]. If only patients with active disease were included, it would not be possible to know whether the biomarker was a marker of the diagnosis or a measure of disease activity. Similarly, we recorded whether both patients with early disease and patients with late disease were included, to determine whether the biomarker was associated with the disease, regardless of stage. We noted whether any disease control group was used to assess the specificity of associations with the biomarker.

#### Studies of biomarkers for disease activity assessment

We examined eight study-design features (Table 1), including whether patients with active and inactive disease were matched for age, sex, and race, or whether a statistical adjustment was made for these factors, and whether the authors reported any one of three possible methods to control for confounding by treatment. We recorded whether changes in the biomarker were assessed longitudinally in some patients. Longitudinal studies provide necessary information on the stability and change of measures with disease activity. We also recorded whether validated activity measures were used, and whether patients with a wide range of disease activity were studied, which would help ensure that tests of associations were adequate.

#### Studies of biomarkers of prognosis assessment

We examined seven design features (Table 1), including age, sex, race matching (or statistical adjustment for these factors), and testing of treatment differences between patients with good or poor prognosis. The use of validated measures of organ damage and longitudinal or cross-sectional design also were evaluated.

### Statistical analysis

We tabulated the proportion of studies that included information on each study-design feature. Proportions were reported separately for studies addressing diagnosis, disease-activity assessment, and prognosis, and for each disease category.

For studies that assessed biomarkers for diagnosis, we examined whether certain study characteristics were associated with whether important design features were more likely to have been included. These characteristics were disease (RA versus other); number of patients; studies with data on humans and animals versus studies that included only data on humans; category of biomarker; measurement of the biomarker on histologic specimens versus other types of specimens; and journal. We first examined these associations in univariate analyses by using χ^2 ^tests. Next we used these characteristics as the independent variables in multivariate logistic regression models that predicted the presence or absence of the study-design features. Each study-design feature was used as the dependent variable in separate models. These models provided adjusted odds ratios that estimated whether, for example, studies that included larger numbers of patients were more or less likely to include age-matched samples, adjusting for disease, inclusion of animal data, category of biomarker, examination of histologic specimens, and journal. We considered 95% confidence intervals of the odds ratio that did not include 1.0 to be statistically significant. The adequacy of model fit was assessed by using the Hosmer-Lemeshow test. Too few studies of disease-activity assessment and prognosis assessment were available to permit a similar analysis. We used SAS programs version 9.2 (SAS Institute, Cary, NC) for all analyses.

## Results

### Results of the literature search

**Figure 1:**
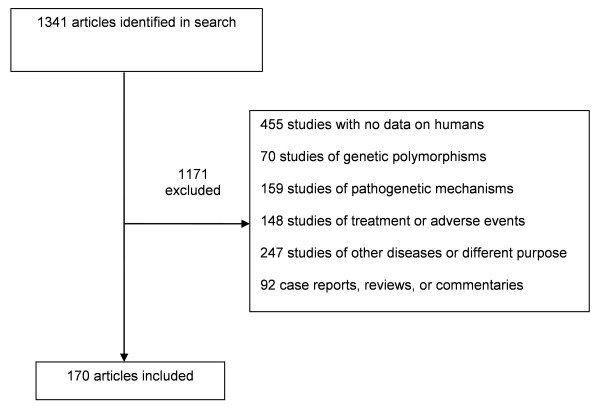
**Results of the literature search and selection**.

We identified 1,341 unique articles. Of these, we excluded 1,171 articles (Figure 1). Among the 170 articles included, 156 articles examined potential biomarkers for diagnosis, 37 articles examined disease activity assessment, and nine articles examined prognosis (articles are listed in the Appendix in Additional file 1). Twenty articles evaluated potential biomarkers for both diagnosis and disease-activity assessment, four for both diagnosis and prognosis, and three for both disease activity and prognosis. Most articles (*n *= 119) were published in *Arthritis and Rheumatism*, 39 in *Journal of Immunology*, five in *Journal of Experimental Medicine*, and seven in the remaining journals. Thirteen studies included data on both humans and animals. The categories of potential biomarkers are shown in Table 2.

**Table 2:** Categories of potential biomarkers examined in translational studies of systemic autoimmune diseases

Biomarkers for diagnosis				
	All diseases	SLE	RA	**Other diseases**^**a**^
	*n *= 156	*n *= 48	*n *= 67	*n *= 41
Categories of biomarkers	*n *(%)	*n *(%)	*n *(%)	*n *(%)
Gene expression	48 (30.8)	9 (18.7)	26 (38.8)	13 (31.7)
Cytokines and receptors	26 (16.7)	6 (12.5)	10 (14.9)	10 (24.4)
Cell-surface markers	23 (14.7)	8 (16.7)	11 (16.4)	4 (9.8)
Lymphocyte markers	20 (12.8)	14 (29.2)	2 (3.0)	4 (9.8)
Chemokines and receptors	11 (7.0)	2 (4.2)	4 (6.0)	5 (12.2)
Miscellaneous^b^	8 (5.1)	1 (2.1)	4 (6.0)	3 (7.3)
Enzymes	6 (3.8)	2 (4.2)	3 (4.5)	1 (2.4)
Endothelial activation markers	5 (3.2)	2 (4.2)	3 (4.5)	0
Soluble cell-surface molecules	4 (2.5)	1 (2.1)	2 (3.0)	1 (2.4)
Signaling molecules	3 (1.9)	2 (4.2)	1 (1.5)	0
Proteomes	2 (1.3)	1 (2.1)	1 (1.4)	0

				
**Biomarkers for disease activity**				
	**All diseases**	**SLE**	**RA**	**Other diseases**^**a**^
	***n *= 37**	***n *= 27**	***n *= 9**	***n *= 1**
	***n *(%)**	***n *(%)**	***n *(%)**	***n *(%)**

Gene expression	10 (27.0)	7 (25.9)	2 (22.2)	1 (100.0)
Lymphocyte markers	8 (21.6)	7 (25.9)	0	0
Cell-surface markers	8 (21.6)	8 (29.6)	1 (11.1)	0
Cytokines and receptors	4 (10.8)	1 (3.7)	3 (33.3)	0
Chemokines and receptors	4 (10.8)	1 (3.7)	3 (33.3)	0
Soluble cell-surface molecules	2 (5.4)	2 (7.4)	0	0
Proteomes	1 (2.7)	1 (3.7)	0	0

				
**Biomarkers for prognosis**				
	**All diseases**	**SLE**	**RA**	**Other diseases**^**a**^
	***n *= 9**	***n *= 3**	***n *= 5**	***n *= 1**
	***n *(%)**	***n *(%)**	***n *(%)**	***n *(%)**

Cytokines and receptors	2 (22.2)	0	2 (50.0)	0
Chemokines and receptors	2 (22.2)	1 (33.3)	0	1 (100.0)
Gene expression	2 (22.2)	1 (33.3)	1 (25.0)	0
Miscellaneous^b^	2 (22.2)	1 (33.3)	1 (25.0)	
Proteomes	1 (11.1)	0	1 (25.0)	0

RA, rheumatoid arthritis; SLE, systemic lupus erythematosus.

^a^Juvenile idiopathic arthritis, Sjögren syndrome, anti-neutrophil cytoplasmic antibody-associated vasculitis, and inflammatory myopathies.

^b^Miscellaneous: uncommonly studied biomarkers such as antibodies, enzymes, and urinary markers.

### Studies of biomarkers for diagnosis

The median number of patients in these studies was 22 (range, 3 to 204), and the median number of controls was 15 (range, 1 to 260). Gene-expression profile was the most frequent category of biomarker, followed by cytokines or cytokine receptors or both, cell-surface markers, and lymphocyte markers. In 36% of studies, the biomarker measurements were based on histologic specimens.

Fewer than one-half of studies included groups that were age and sex matched to controls, provided information on medications, or included both patients with active disease and patients with inactive disease (Table 3). For example, 47.4% of studies had age-matched groups, and 46.1% had sex-matched groups. Moreover, 44 (28.2%) studies did not report any information on age, 47 (30.1%) studies did not report sex, and 102 (65.4%) studies did not report race. Only 35.3% of studies reported any of the three approaches to control for treatment differences. Fewer studies in RA included these design features than did studies in SLE or other diseases, and 22.4% of RA studies did not report whether patients met accepted classification criteria.

**Table 3:** Proportion of studies examining potential biomarkers for diagnosis that incorporated important study-design features

	All diseases	SLE	RA	**Other diseases**^**a**^
	*n *= 156	*n *= 48	*n *= 67	*n *= 41
Study-design features	*n *(%)	*n *(%)	*n *(%)	*n *(%)
Matched for age	74 (47.4)	30 (62.5)	23 (34.3)	21 (51.2)
Matched for sex	72 (46.1)	28 (58.3)	24 (35.8)	20 (48.8)
Matched for race	51 (32.7)	23 (47.9)	20 (29.8)	8 (19.5)
Provided information on medications	79 (50.6)	34 (70.8)	25 (37.3)	20 (48.8)
Controlled for treatment effects	55 (35.3)	25 (52.0)	13 (19.4)	17 (41.4)
Reported use of accepted classification criteria	140 (89.7)	48 (100.0)	52 (77.6)	40 (97.6)
Included patients with both early and late disease	43 (27.6)	16 (33.0)	18 (26.9)	9 (21.9)
Included patients with both active and inactive disease	58 (43.3)	40 (83.3)	15 (22.4)	3 (15.8)
Included disease controls	61 (39.1)	27 (56.2)	16 (23.9)	18 (43.9)

RA, rheumatoid arthritis; SLE, systemic lupus erythematosus.

^a^Juvenile idiopathic arthritis, Sjögren syndrome, anti-neutrophil cytoplasmic antibody-associated vasculitis, and inflammatory myopathies.

Three (1.9%) studies included all nine study-design features, 61.2% of studies included at least four features, but only 18% of studies simultaneously controlled for age, sex, and treatment differences. An example of a study that included all nine design features was the 2006 report by Feng and colleagues [12] of the association between interferon-inducible gene expression and SLE. Expression of a set of five interferon-inducible genes was found to be increased among 48 patients with SLE compared with 48 healthy controls and 22 disease controls matched for age, sex, and ethnicity. The patients with SLE fulfilled current classification criteria, had durations of SLE that ranged from less than 1 year to 37 years, and had scores for SLE activity that ranged from inactive to very active. Interferon scores were not associated with the type or intensity of immunosuppressive treatment, after accounting for SLE activity.

**Figure 2:**
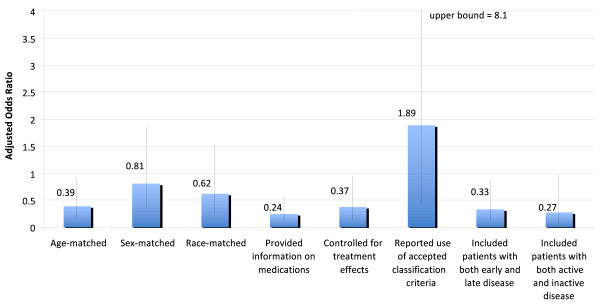
**Associations between studies using histologic specimens (versus other types of specimens) and the presence of each study-design feature, by multivariate logistic regression analysis**. Odds ratios <1.0 indicate that the study-design feature was less common in studies using histologic specimens, whereas odds ratios >1.0 indicate that the study-design feature was more common in studies of histologic specimens. Error bars are 95% confidence limits. Odds ratios were based on models that also included disease (RA versus other), journal, the number of patients, data on animals, and category of biomarker as independent variables. Separate models were estimated for each study-design feature. Each model fit the data well (all Hosmer-Lemeshow test, *P *> 0.09).

**Figure 3:**
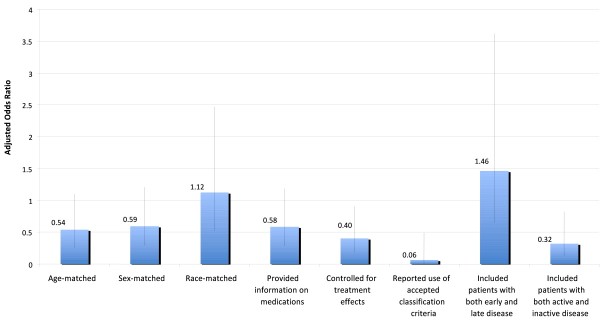
**Associations between studies of RA (versus SLE and other diseases) and the presence of each study-design feature, by multivariate logistic regression analysis**. Odds ratios <1.0 indicate that the study-design feature was less common in studies of RA, whereas odds ratios >1.0 indicate that the study-design feature was more common in studies of RA. Error bars are 95% confidence limits Odds ratios were based on models that also included the type of specimen, journal, the number of patients, data on animals, and category of biomarker as independent variables. Separate models were estimated for each study-design feature. Each model fit the data well (all Hosmer-Lemeshow test, *P *> 0.09).

We examined whether selected study characteristics were associated with the likelihood that important study-design features had been reported. In univariate analyses, studies with smaller numbers of subjects, studies of RA, and studies that examined histologic specimens were less likely to include these study-design features. Studies using histologic specimens were smaller (median number of patients, 10) than other studies (median number, 36), and most often examined RA (55.3%). To distinguish which of these factors were independently associated with the likelihood that each study-design feature had been included, we used multivariate logistic regression analysis. In these analyses, studies that measured biomarkers on histologic specimens were significantly less likely to include age-matched groups, report the medications used by patients, control for treatment differences, include patients with both early and late disease, and include patients with both active and inactive disease (Figure 2). Studies of RA were significantly less likely than studies of other diseases to control for treatment effects, report using accepted classification criteria, and include patients with both active and inactive disease (Figure 3). Larger studies were more likely to include sex-matched groups (adjusted odds ratio (aOR) (per each additional 10 patients), 1.16; 95% confidence interval (CI), 1.03 to 1.30), race-matched groups (aOR, 1.18; 95% CI, 1.03 to 1.36), and patients with both active and inactive disease (aOR, 1.30; 95% CI, 1.08 to 1.57).

### Biomarkers for disease-activity assessment

The median number of patients was 45 (range, 9 to 245). Only one third of studies adjusted for group differences in age and sex; 56.8% reported at least one method to control for treatment effects; and 45.9% had a longitudinal component (Table 4). Studies in RA were less likely to report adjustment for age and sex and to control for treatment effects, than were studies in SLE. No studies that examined histologic specimens reported that groups were adjusted for age, sex, or race, whereas 31.4%, 34%, and 45.7%, respectively, of studies that examined other types of specimens did so.

**Table 4:** Proportion of studies examining potential biomarkers for disease activity that incorporated important study-design features

	All diseases	SLE	RA	**Other diseases**^**a**^
	*n *= 37	*n *= 27	*n *= 9	*n *= 1
Study-design features	*n *(%)	*n *(%)	*n *(%)	*n *(%)
Adjusted for age	11 (29.7)	10 (37.0)	1 (11.1)	0
Adjusted for sex	12 (32.4)	11 (40.7)	1 (11.1)	0
Adjusted for race	16 (43.2)	11 (40.7)	4 (44.4)	1 (100.0)
Provided information on medications	23 (62.1)	18 (66.7)	4 (44.4)	1 (100.0)
Controlled for treatment effects	21 (56.8)	16 (59.2)	4 (44.4)	0
Longitudinal component	17 (45.9)	11 (40.7)	5 (55.6)	1 (100.0)
Validated disease-activity measures	34 (91.9)	27 (100.0)	7 (77.8)	0
Included wide range of disease activity	34 (91.9)	24 (88.9)	9 (100.0)	1 (100.0)

RA, rheumatoid arthritis; SLE, systemic lupus erythematosus.

^a^Juvenile idiopathic arthritis, Sjögren syndrome, anti-neutrophil cytoplasmic antibody-associated vasculitis, and inflammatory myopathies.

### Biomarkers for prognosis assessment

The median number of patients was 60 (range, 15 to 92). Few studies adjusted for group differences in age (44.4%) or sex (22.2%), or had a longitudinal component (33.3%). Two thirds of studies controlled for treatment effects, and all reported using validated outcome measures.

## Discussion

The diagnosis, monitoring, and management of patients with systemic autoimmune diseases remain challenging, prompting a continuing search for better biomarkers. In the progression from exploratory to validated status, biomarkers must undergo careful evaluation of sources of variation [5-7,13-15]. We found that fewer than one half of translational studies of biomarkers included study-design features needed for valid interpretation of clinical associations.

Gene-expression profiles were the most common potential biomarkers. If the patient and control groups are not comparable in biologic factors that might influence gene expression, the association of a particular set of genes with disease may be mistaken, whereas an unrecognized set of genes may be the true disease-associated set. Two large-scale surveys of variation in gene expression in healthy subjects showed substantial differences within individuals over time and among individuals by age and sex [16,17]. Immune-related genes, including those for immunoglobulins and genes that are regulated by interferons, were among the genes most often found to be differentially expressed by age and sex [16,17]. This is particularly relevant because patterns of expression of specific type 1 interferon-regulated genes, so-called "interferon signatures," have been proposed as useful biomarkers in SLE and inflammatory myopathies [18,19]. Moreover, if gene expression is differentially regulated by medications, misidentification of treatment-responsive genes as disease-related signatures may occur. Similar effects can occur for other categories of biomarkers.

Studies of biomarkers of disease activity have an additional limitation if they are examined only cross-sectionally. Cross-sectional studies compare expression of the biomarker between patients with active and inactive disease, without evidence that expression can change as disease activity changes. Longitudinal studies of patients who experience changes in activity provide a more valid design [5,6]. Fewer than one half of studies included a longitudinal design.

Most studies showed the potential biomarker to be positively associated with the disease investigated, despite often-limited attention to confounding. Of greater concern is the possibility that other studies failed to detect associations because of inattention to these issues. The failure to consider matching, treatment effects, and other study-design features might have led investigators to conclude that no association was present, and therefore, to abandon potentially promising biomarkers. False-negative results may be common, but because negative studies may not be reported, the extent of this problem is difficult to assess.

Studies based on histologic specimens and studies of RA were less likely to address important study-design features than were studies based on serum or other sources and studies of SLE or other diseases, respectively. Although studies of histologic specimens were generally of smaller size, and most often were studies of the synovium in RA, the associations were independent of both sample size and disease. Attention to study-design features may be less prevalent among these studies because histologic samples are more difficult to obtain, and specimens are used when they become available. In studies of animal models, age, sex, and genetic background are considered important factors to be controlled. Human studies should similarly consider these factors. Larger studies, likely representing biomarkers at a more advanced stage of evaluation, more often reported important study-design features.

Our results reflect existing community standards for studies in systemic autoimmune diseases. The deficiencies may be due in part to incomplete reporting rather than to omissions in the study design. Adoption of uniform reporting criteria would be one remedy for this problem. To the extent that these deficiencies are due to omissions in study design, our results raise questions of whether attention to issues in the testing of clinical applications of the biomarker is often overshadowed by the novelty of the laboratory science.

The strengths of this study include examination of a diverse set of potential biomarkers in several diseases, many study-design features, and three clinical applications. However, our conclusions about biomarkers for prognosis are limited by the small number of studies. Inclusion of additional years or journals might have increased the number of studies, but our search provided a broad representation of recent studies. Some study-design features, such as adjustment for age, sex, and treatment effects, may be considered more important than others, but we examined a broader set of features without suggesting a hierarchy among them. The proportion of studies that included age, sex, or race adjustment was low, even though our criteria for matching were liberal. Although we examined only systemic autoimmune diseases, similar results may occur in other diseases such as osteoarthritis, spondyloarthritis, cancer, or cardiovascular disease [20-25].

## Conclusions

Many early studies of potential biomarkers in systemic autoimmune diseases did not include design features important for valid clinical associations. Greater attention to the design of translational studies of biomarkers would result in more-accurate assessments of their potential clinical applications and reduce false-positive and false-negative associations. Researchers should include adjustment for differences in demographic characteristics and test whether commonly used treatments affect the biomarker. Studies of biomarkers of disease activity should include a longitudinal component. Readers of studies of new biomarkers should assess whether these study-design features were included. Biomarker discovery may benefit from greater involvement by clinical epidemiologists in the design of early translational studies.

## Abbreviations

aOR: adjusted odds ratio; RA: rheumatoid arthritis; SLE: systemic lupus erythematosus.

## Competing interests

The authors declare that they have no competing interests.

## Authors' contributions

MGT and MMW designed the study. MGT performed the literature search. MGT and MMW abstracted the data. MMW performed the statistical analysis. MGT drafted the manuscript. MMW participated in revising the draft. Both authors approved the final version.

## Supplementary Material

Additional file 1 **Appendix**. Reference list of articles included in the study. Click here for file
