# Supplementary material for: Validity of clinical associations of biomarkers in translational research studies: the case of systemic autoimmune diseases
Source: Arthritis Res Ther. 2010 Sep 27;12(5):R179. doi: 10.1186/ar3143 (PMC2991010; doi:10.1186/ar3143)
Supplement: Additional file 1 — Appendix. Reference list of articles included in the study. [file ar3143-S1.DOC]

**Appendix**

1. Dai Z, Turtle CJ, Booth GC, Riddell SR, Gooley TA, Stevens AM, et al. Normally occurring NKG2D+CD4+ T cells are immunosuppressive and inversely correlated with disease activity in juvenile-onset lupus. J Exp Med. 2009;206:793-805.
2. Mackay M, Stanevsky A, Wang T, Aranow C, Li M, Koenig S, et al. Selective dysregulation of the FcgammaIIB receptor on memory B cells in SLE. J Exp Med. 2006;203:2157-64.
3. Katsiari CG, Kyttaris VC, Juang YT, Tsokos GC. Protein phosphatase 2A is a negative regulator of IL-2 production in patients with systemic lupus erythematosus. J Clin Invest. 2005;115:3193-204.
4. Li QZ, Xie C, Wu T, Mackay M, Aranow C, Putterman C, et al. Identification of autoantibody clusters that best predict lupus disease activity using glomerular proteome arrays. J Clin Invest. 2005;115:3428-39.
5. Santer DM, Yoshio T, Minota S, Möller T, Elkon KB. Potent induction of IFN-alpha and chemokines by autoantibodies in the cerebrospinal fluid of patients with neuropsychiatric lupus. J Immunol. 2009;182:1192-201.
6. Garaud S, Le Dantec C, Jousse-Joulin S, Hanrotel-Saliou C, Saraux A, Mageed RA, et al. IL-6 modulates CD5 expression in B cells from patients with lupus by regulating DNA methylation. J Immunol. 2009;182:5623-32.
7. Crispín JC, Oukka M, Bayliss G, Cohen RA, Van Beek CA, Stillman IE, et al. Expanded double negative T cells in patients with systemic lupus erythematosus produce IL-17 and infiltrate the kidneys. J Immunol. 2008;181:8761-6.
8. Harigai M, Kawamoto M, Hara M, Kubota T, Kamatani N, Miyasaka N. Excessive production of IFN-gamma in patients with systemic lupus erythematosus and its contribution to induction of B lymphocyte stimulator/B cell-activating factor/TNF ligand superfamily-13B. J Immunol. 2008;181:2211-9.
9. Chang NH, McKenzie T, Bonventi G, Landolt-Marticorena C, Fortin PR, Gladman D, et al. Expanded population of activated antigen-engaged cells within the naive B cell compartment of patients with systemic lupus erythematosus. J Immunol. 2008;180:1276-84.
10. Jury EC, Eldridge J, Isenberg DA, Kabouridis PS. Agrin signalling contributes to cell activation and is overexpressed in T lymphocytes from lupus patients. J Immunol. 2007;179:7975-83.
11. Wu T, Xie C, Wang HW, Zhou XJ, Schwartz N, Calixto S, et al. Elevated urinary VCAM-1, P-selectin, soluble TNF receptor-1, and CXC chemokine ligand 16 in multiple murine lupus strains and human lupus nephritis. J Immunol. 2007;179:7166-75.
12. Valencia X, Yarboro C, Illei G, Lipsky PE. Deficient CD4+CD25high T regulatory cell function in patients with active systemic lupus erythematosus. J Immunol. 2007;178:2579-88.
13. Kyttaris VC, Wang Y, Juang YT, Weinstein A, Tsokos GC. Increased levels of NF-ATc2 differentially regulate CD154 and IL-2 genes in T cells from patients with systemic lupus erythematosus. J Immunol. 2007;178:1960-6.
14. Ding D, Mehta H, McCune WJ, Kaplan MJ. Aberrant phenotype and function of myeloid dendritic cells in systemic lupus erythematosus. J Immunol. 2006;177:5878-89.
15. Krishnan S, Juang YT, Chowdhury B, Magilavy A, Fisher CU, Nguyen H, et al. Differential expression and molecular associations of Syk in systemic lupus erythematosus T cells. J Immunol. 2008;181:8145-52.
16. Krishnan S, Kiang JG, Fisher CU, Nambiar MP, Nguyen HT, Kyttaris VC, et al. Increased caspase-3 expression and activity contribute to reduced CD3zeta expression in systemic lupus erythematosus T cells. J Immunol. 2005;175:3417-23.
17. Wei C, Anolik J, Cappione A, Zheng B, Pugh-Bernard A, Brooks J, et al. A new population of cells lacking expression of CD27 represents a notable component of the B cell memory compartment in systemic lupus erythematosus. J Immunol. 2007;178:6624-33.
18. Tao D, Shangwu L, Qun W, Yan L, Wei J, Junyan L, et al. CD226 expression deficiency causes high sensitivity to apoptosis in NK T cells from patients with systemic lupus erythematosus. J Immunol. 2005;174:1281-90.
19. Lee J, Kuchen S, Fischer R, Chang S, Lipsky PE. Identification and characterization of a human CD5+ pre-naive B cell population. J Immunol. 2009;182:4116-26.
20. Su K, Yang H, Li X, Li X, Gibson AW, Cafardi JM, et al. Expression profile of FcgammaRIIb on leukocytes and its dysregulation in systemic lupus erythematosus. J Immunol. 2007;178:3272-80.
21. Miyara M, Amoura Z, Parizot C, Badoual C, Dorgham K, Trad S, et al. Global natural regulatory T cell depletion in active systemic lupus erythematosus. J Immunol. 2005;175:8392-400.
22. Hutcheson J, Scatizzi JC, Siddiqui AM, Haines GK 3rd, Wu T, Li QZ, et al. Combined deficiency of proapoptotic regulators Bim and Fas results in the early onset of systemic autoimmunity. Immunity. 2008;28:206-17.
23. Doreau A, Belot A, Bastid J, Riche B, Trescol-Biemont MC, Ranchin B, et al. Interleukin 17 acts in synergy with B cell-activating factor to influence B cell biology and the pathophysiology of systemic lupus erythematosus. Nat Immunol. 2009;10:778-85.
24. Lee PY, Li Y, Richards HB, Chan FS, Zhuang H, Narain S, et al. Type I interferon as a novel risk factor for endothelial progenitor cell depletion and endothelial dysfunction in systemic lupus erythematosus. Arthritis Rheum. 2007;56:3759-69.
25. Nakayamada S, Saito K, Nakano K, Tanaka Y. Activation signal transduction by beta1 integrin in T cells from patients with systemic lupus erythematosus. Arthritis Rheum. 2007;56:1559-68.
26. Fragoso-Loyo H, Richaud-Patin Y, Orozco-Narváez A, Dávila-Maldonado L, Atisha-Fregoso Y, Llorente L, et al. Interleukin-6 and chemokines in the neuropsychiatric manifestations of systemic lupus erythematosus. Arthritis Rheum. 2007;56:1242-50.
27. Oates JC, Farrelly LW, Hofbauer AF, Wang W, Gilkeson GS. Association of reactive oxygen and nitrogen intermediate and complement levels with apoptosis of peripheral blood mononuclear cells in lupus patients. Arthritis Rheum. 2007;56:3738-47.
28. Hua J, Kirou K, Lee C, Crow MK. Functional assay of type I interferon in systemic lupus erythematosus plasma and association with anti-RNA binding protein autoantibodies. Arthritis Rheum. 2006;54:1906-16.
29. Tucci M, Quatraro C, Lombardi L, Pellegrino C, Dammacco F, Silvestris F. Glomerular accumulation of plasmacytoid dendritic cells in active lupus nephritis: role of interleukin-18.Arthritis Rheum. 2008;58:251-62.
30. Petri M, Stohl W, Chatham W, McCune WJ, Chevrier M, Ryel J, et al. Association of plasma B lymphocyte stimulator levels and disease activity in systemic lupus erythematosus. Arthritis Rheum. 2008;58:2453-9.
31. Yan B, Ye S, Chen G, Kuang M, Shen N, Chen S. Dysfunctional CD4+,CD25+ regulatory T cells in untreated active systemic lupus erythematosus secondary to interferon-alpha-producing antigen-presenting cells. Arthritis Rheum. 2008;58:801-12.
32. Tang Y, Luo X, Cui H, Ni X, Yuan M, Guo Y, et al. MicroRNA-146A contributes to abnormal activation of the type I interferon pathway in human lupus by targeting the key signaling proteins. Arthritis Rheum. 2009;60:1065-75.
33. Enghard P, Humrich JY, Rudolph B, Rosenberger S, Biesen R, Kuhn A, et al. CXCR3+CD4+ T cells are enriched in inflamed kidneys and urine and provide a new biomarker for acute nephritis flares in systemic lupus erythematosus patients. Arthritis Rheum. 2009;60:199-206.
34. Brunner HI, Mueller M, Rutherford C, Passo MH, Witte D, Grom A, et al. Urinary neutrophil gelatinase-associated lipocalin as a biomarker of nephritis in childhood-onset systemic lupus erythematosus. Arthritis Rheum. 2006;54:2577-84.
35. Pitashny M, Schwartz N, Qing X, Hojaili B, Aranow C, Mackay M, et al. Urinary lipocalin-2 is associated with renal disease activity in human lupus nephritis. Arthritis Rheum. 2007;56:1894-903.
36. Liu CC, Manzi S, Kao AH, Navratil JS, Ruffing MJ, Ahearn JM. Reticulocytes bearing C4d as biomarkers of disease activity for systemic lupus erythematosus. Arthritis Rheum. 2005;52:3087-99.
37. Navratil JS, Manzi S, Kao AH, Krishnaswami S, Liu CC, Ruffing MJ, et al. Platelet C4d is highly specific for systemic lupus erythematosus. Arthritis Rheum. 2006;54:670-4.
38. Biesen R, Demir C, Barkhudarova F, Grün JR, Steinbrich-Zöllner M, Backhaus M, et al. Sialic acid-binding Ig-like lectin 1 expression in inflammatory and resident monocytes is a potential biomarker for monitoring disease activity and success of therapy in systemic lupus erythematosus. Arthritis Rheum. 2008;58:1136-45.
39. Feng X, Wu H, Grossman JM, Hanvivadhanakul P, FitzGerald JD, Park GS, et al. Association of increased interferon-inducible gene expression with disease activity and lupus nephritis in patients with systemic lupus erythematosus. Arthritis Rheum. 2006;54:2951-62.
40. Yang J, Chu Y, Yang X, Gao D, Zhu L, Yang X, et al. Th17 and natural Treg cell population dynamics in systemic lupus erythematosus. Arthritis Rheum. 2009;60:1472-83.
41. Flores-Borja F, Kabouridis PS, Jury EC, Isenberg DA, Mageed RA. Altered lipid raft-associated proximal signaling and translocation of CD45 tyrosine phosphatase in B lymphocytes from patients with systemic lupus erythematosus. Arthritis Rheum. 2007;56:291-302.
42. Park YW, Kee SJ, Cho YN, Lee EH, Lee HY, Kim EM, et al. Impaired differentiation and cytotoxicity of natural killer cells in systemic lupus erythematosus. Arthritis Rheum. 2009;60:1753-63.
43. Chan RW, Lai FM, Li EK, Tam LS, Wong TY, Szeto CY, et al. Expression of chemokine and fibrosing factor messenger RNA in the urinary sediment of patients with lupus nephritis. Arthritis Rheum. 2004;50:2882-90.
44. Kirou KA, Lee C, George S, Louca K, Papagiannis IG, Peterson MG, et al. Coordinate overexpression of interferon-alpha-induced genes in systemic lupus erythematosus. Arthritis Rheum. 2004;50:3958-67.
45. Fritsch RD, Shen X, Illei GG, Yarboro CH, Prussin C, Hathcock KS, et al. Abnormal differentiation of memory T cells in systemic lupus erythematosus.Arthritis Rheum. 2006;54:2184-97.
46. Jacobi AM, Reiter K, Mackay M, Aranow C, Hiepe F, Radbruch A, et al. Activated memory B cell subsets correlate with disease activity in systemic lupus erythematosus: delineation by expression of CD27, IgD, and CD95. Arthritis Rheum. 2008;58:1762-73.
47. Ripley BJ, Rahman MA, Isenberg DA, Latchman DS. Elevated expression of the Brn-3a and Brn-3b transcription factors in systemic lupus erythematosus correlates with antibodies to Brn-3 and overexpression of Hsp90. Arthritis Rheum. 2005;52:1171-9.
48. Green MR, Kennell AS, Larche MJ, Seifert MH, Isenberg DA, Salaman MR. Natural killer T cells in families of patients with systemic lupus erythematosus: their possible role in regulation of IGG production. Arthritis Rheum. 2007;56:303-10.
49. Carter RH, Zhao H, Liu X, Pelletier M, Chatham W, Kimberly R, et al. Expression and occupancy of BAFF-R on B cells in systemic lupus erythematosus. Arthritis Rheum. 2005 Dec;52(12):3943-54.
50. Kirou KA, Lee C, George S, Louca K, Peterson MG, Crow MK. Activation of the interferon-alpha pathway identifies a subgroup of systemic lupus erythematosus patients with distinct serologic features and active disease. Arthritis Rheum. 2005;52:1491-503.
51. Yajima N, Kasama T, Isozaki T, Odai T, Matsunawa M, Negishi M, et al. Elevated levels of soluble fractalkine in active systemic lupus erythematosus: potential involvement in neuropsychiatric manifestations. Arthritis Rheum. 2005;52:1670-5.
52. Okamoto H, Katsumata Y, Nishimura K, Kamatani N. Interferon-inducible protein 10/CXCL10 is increased in the cerebrospinal fluid of patients with central nervous system lupus. Arthritis Rheum. 2004;50:3731-2.
53. Nzeusseu Toukap A, Galant C, Theate I, Maudoux AL, Lories RJ, Houssiau FA, et al. Identification of distinct gene expression profiles in the synovium of patients with systemic lupus erythematosus. Arthritis Rheum. 2007;56:1579-88.
54. Manzi S, Navratil JS, Ruffing MJ, Liu CC, Danchenko N, Nilson SE, et al.Measurement of erythrocyte C4d and complement receptor 1 in systemic lupus erythematosus. Arthritis Rheum. 2004;50:3596-604.
55. Pascual V, Allantaz F, Arce E, Punaro M, Banchereau J. Role of interleukin-1 (IL-1) in the pathogenesis of systemic onset juvenile idiopathic arthritis and clinical response to IL-1 blockade. J Exp Med. 2005;201:1479-86.
56. Samuels J, Ng YS, Coupillaud C, Paget D, Meffre E. Impaired early B cell tolerance in patients with rheumatoid arthritis. J Exp Med. 2005;201:1659-67.
57. Allantaz F, Chaussabel D, Stichweh D, Bennett L, Allman W, Mejias A, et al. Blood leukocyte microarrays to diagnose systemic onset juvenile idiopathic arthritis and follow the response to IL-1 blockade. J Exp Med. 2007;204:2131-44.
58. Xu G, Nie H, Li N, Zheng W, Zhang D, Feng G, et al. Role of osteopontin in amplification and perpetuation of rheumatoid synovitis. J Clin Invest. 2005;115:1060-7.
59. Takagi R, Higashi T, Hashimoto K, Nakano K, Mizuno Y, Okazaki Y, et al. B cell chemoattractant CXCL13 is preferentially expressed by human Th17 cell clones. J Immunol. 2008;181:186-9.
60. Witkowski JM, Soroczyńska-Cybula M, Bryl E, Smoleńska Z, Jóźwik A. Klotho--a common link in physiological and rheumatoid arthritis-related aging of human CD4+ lymphocytes. Immunol. 2007;178:771-7.
61. V, Haines GK 3rd, Perlman H, Koch AE, et al. Mcl-1 is essential for the survival of synovial fibroblasts in rheumatoid arthritis.J Immunol. 2005;175:8337-45.
62. Bokarewa M, Nagaev I, Dahlberg L, Smith U, Tarkowski A. Resistin, an adipokine with potent proinflammatory properties. J Immunol. 2005;174:5789-95.
63. Lande R, Giacomini E, Serafini B, Rosicarelli B, Sebastiani GD, Minisola G, et al. Characterization and recruitment of plasmacytoid dendritic cells in synovial fluid and tissue of patients with chronic inflammatory arthritis. J Immunol. 2004;173:2815-24.
64. Uzan B, Ea HK, Launay JM, Garel JM, Champy R, Cressent M, et al. A critical role for adrenomedullin-calcitonin receptor-like receptor in regulating rheumatoid fibroblast-like synoviocyte apoptosis. J Immunol. 2006;176:5548-58.
65. Kimura M, Kawahito Y, Obayashi H, Ohta M, Hara H, Adachi T, et al. A critical role for allograft inflammatory factor-1 in the pathogenesis of rheumatoid arthritis.J Immunol. 2007;178:3316-22.
66. Wan B, Nie H, Liu A, Feng G, He D, Xu R, et al. Aberrant regulation of synovial T cell activation by soluble costimulatory molecules in rheumatoid arthritis. Immunol. 2006;177:8844-50.
67. Zhu L, Ji F, Wang Y, Zhang Y, Liu Q, Zhang JZ, et al. Synovial autoreactive T cells in rheumatoid arthritis resist IDO-mediated inhibition. J Immunol. 2006;177:8226-33.
68. Devauchelle V, Essabbani A, De Pinieux G, Germain S, Tourneur L, Mistou S, et al. Characterization and functional consequences of underexpression of clusterin in rheumatoid arthritis. Immunol. 2006;177:6471-9.
69. Singh K, Colmegna I, He X, Weyand CM, Goronzy JJ. Synoviocyte stimulation by the LFA-1-intercellular adhesion molecule-2-Ezrin-Akt pathway in rheumatoid arthritis. J Immunol. 2008 1;180:1971-8.
70. Zrioual S, Toh ML, Tournadre A, Zhou Y, Cazalis MA, Pachot A, Miossec V, Miossec P.IL-17RA and IL-17RC receptors are essential for IL-17A-induced ELR+ CXC chemokine expression in synoviocytes and are overexpressed in rheumatoid blood. J Immunol. 2008;180:655-63.
71. Zrioual S, Ecochard R, Tournadre A, Lenief V, Cazalis MA, Miossec P. Genome-wide comparison between IL-17A- and IL-17F-induced effects in human rheumatoid arthritis synoviocytes. J Immunol. 2009;182:3112-20.
72. Lee WW, Yang ZZ, Li G, Weyand CM, Goronzy JJ. Unchecked CD70 expression on T cells lowers threshold for T cell activation in rheumatoid arthritis. J Immunol. 2007;179:2609-15.
73. Cassatella MA, Pereira-da-Silva G, Tinazzi I, Facchetti F, Scapini P, Calzetti F, et al. Soluble TNF-like cytokine (TL1A) production by immune complexes stimulated monocytes in rheumatoid arthritis. J Immunol. 2007;178:7325-33.
74. Ohata J, Zvaifler NJ, Nishio M, Boyle DL, Kalled SL, Carson DA, et al. Fibroblast-like synoviocytes of mesenchymal origin express functional B cell-activating factor of the TNF family in response to proinflammatory cytokines. J Immunol. 2005174:864-70.
75. Kim WU, Kang SS, Yoo SA, Hong KH, Bae DG, Lee MS, et al. Interaction of vascular endothelial growth factor 165 with neuropilin-1 protects rheumatoid synoviocytes from apoptotic death by regulating Bcl-2 expression and Bax translocation. J Immunol. 2006;177:5727-35.
76. Di Girolamo N, Indoh I, Jackson N, Wakefield D, McNeil HP, Yan W, et al. Human mast cell-derived gelatinase B (matrix metalloproteinase-9) is regulated by inflammatory cytokines: role in cell migration. J Immunol. 2006 Aug;177:2638-50.
77. Diarra D, Stolina M, Polzer K, Zwerina J, Ominsky MS, Dwyer D, et al. Dickkopf-1 is a master regulator of joint remodeling. Nat Med. 2007;13:156-63.
78. Zheng W, Li R, Pan H, He D, Xu R, Guo TB, et al. Role of osteopontin in induction of monocyte chemoattractant protein 1 and macrophage inflammatory protein 1beta through the NF-kappaB and MAPK pathways in rheumatoid arthritis. Arthritis Rheum. 2009;60:1957-65.
79. Mun SH, Kim JW, Nah SS, Ko NY, Lee JH, Kim JD, et al. Tumor necrosis factor alpha-induced interleukin-32 is positively regulated via the Syk/protein kinase Cdelta/JNK pathway in rheumatoid synovial fibroblasts. Arthritis Rheum. 2009;60:678-85.
80. Rabquer BJ, Pakozdi A, Michel JE, Gujar BS, Haines GK 3rd, Imhof BA, et al. Junctional adhesion molecule C mediates leukocyte adhesion to rheumatoid arthritis synovium. Arthritis Rheum. 2008;58:3020-9.
81. Kop EN, Kwakkenbos MJ, Teske GJ, Kraan MC, Smeets TJ, Stacey M, et al. Identification of the epidermal growth factor-TM7 receptor EMR2 and its ligand dermatan sulfate in rheumatoid synovial tissue.Arthritis Rheum. 2005;52:442-50.
82. de Jong H, Lafeber FF, de Jager W, Haverkamp MH, Kuis W, Bijlsma JW, et al. PAN-DR-Binding Hsp60 self epitopes induce an interleukin-10-mediated immune response in rheumatoid arthritis. Arthritis Rheum. 2009;60:1966-76.
83. Liu M, Sun H, Wang X, Koike T, Mishima H, Ikeda K, et al. Association of increased expression of macrophage elastase (matrix metalloproteinase 12) with rheumatoid arthritis. Arthritis Rheum. 2004;50:3112-7.
84. Pi X, Tan SY, Hayes M, Xiao L, Shayman JA, Ling S, et al. Sphingosine kinase 1-mediated inhibition of Fas death signaling in rheumatoid arthritis B lymphoblastoid cells. Arthritis Rheum. 2006;54:754-64.
85. Pohlers D, Huber R, Ukena B, Kinne RW. Expression of platelet-derived growth factors C and D in the synovial membrane of patients with rheumatoid arthritis and osteoarthritis. Arthritis Rheum. 2006;54:788-94.
86. van der Heijden JW, Oerlemans R, Dijkmans BA, Qi H, van der Laken CJ, Lems WF, et al. Folate receptor beta as a potential delivery route for novel folate antagonists to macrophages in the synovial tissue of rheumatoid arthritis patients. Arthritis Rheum. 2009;60:12-21.
87. Korb A, Tohidast-Akrad M, Cetin E, Axmann R, Smolen J, Schett G. Differential tissue expression and activation of p38 MAPK alpha, beta, gamma, and delta isoforms in rheumatoid arthritis. Arthritis Rheum. 2006;54:2745-56.
88. van Roon JA, Verweij MC, Wijk MW, Jacobs KM, Bijlsma JW, Lafeber FP. Increased intraarticular interleukin-7 in rheumatoid arthritis patients stimulates cell contact-dependent activation of CD4(+) T cells and macrophages. Arthritis Rheum. 2005;52:1700-10.
89. Jarvis JN, Jiang K, Frank MB, Knowlton N, Aggarwal A, Wallace CA, et al. Gene expression profiling in neutrophils from children with polyarticular juvenile idiopathic arthritis. Arthritis Rheum. 2009;60:1488-95.
90. Barnes MG, Grom AA, Thompson SD, Griffin TA, Pavlidis P, Itert L, et al. Subtype-specific peripheral blood gene expression profiles in recent-onset juvenile idiopathic arthritis. Arthritis Rheum. 2009;60:2102-12.
91. Nile CJ, Read RC, Akil M, Duff GW, Wilson AG. Methylation status of a single CpG site in the IL6 promoter is related to IL6 messenger RNA levels and rheumatoid arthritis. Arthritis Rheum. 2008;58:2686-93.
92. Fall N, Barnes M, Thornton S, Luyrink L, Olson J, Ilowite NT, et al. Gene expression profiling of peripheral blood from patients with untreated new-onset systemic juvenile idiopathic arthritis reveals molecular heterogeneity that may predict macrophage activation syndrome. Arthritis Rheum. 2007;56:3793-804.
93. Kitano M, Hla T, Sekiguchi M, Kawahito Y, Yoshimura R, Miyazawa K, et al. Sphingosine 1-phosphate/sphingosine 1-phosphate receptor 1 signaling in rheumatoid synovium: regulation of synovial proliferation and inflammatory gene expression. Arthritis Rheum. 2006;54:742-53.
94. Antoniv TT, Ivashkiv LB. Dysregulation of interleukin-10-dependent gene expression in rheumatoid arthritis synovial macrophages.Arthritis Rheum. 2006;54:2711-21.
95. Ogilvie EM, Khan A, Hubank M, Kellam P, Woo P. Specific gene expression profiles in systemic juvenile idiopathic arthritis. Arthritis Rheum. 2007;56:1954-65.
96. Li RW, Freeman C, Yu D, Hindmarsh EJ, Tymms KE, Parish CR, et al. Dramatic regulation of heparanase activity and angiogenesis gene expression in synovium from patients with rheumatoid arthritis. Arthritis Rheum. 2008;58:1590-600.
97. Prelog M, Schwarzenbrunner N, Sailer-Höck M, Kern H, Klein-Franke A, Ausserlechner MJ, et al. Premature aging of the immune system in children with juvenile idiopathic arthritis. Arthritis Rheum. 2008;58:2153-62.
98. Yoo SA, Yoon HJ, Kim HS, Chae CB, De Falco S, Cho CS, et al. Role of placenta growth factor and its receptor flt-1 in rheumatoid inflammation: a link between angiogenesis and inflammation. Arthritis Rheum. 2009;60:345-54.
99. Foulquier C, Sebbag M, Clavel C, Chapuy-Regaud S, Al Badine R, Méchin MC, et al. Peptidyl arginine deiminase type 2 (PAD-2) and PAD-4 but not PAD-1, PAD-3, and PAD-6 are expressed in rheumatoid arthritis synovium in close association with tissue inflammation. Arthritis Rheum. 2007;56:3541-53.
100. Flood S, Parri R, Williams A, Duance V, Mason D. Modulation of interleukin-6 and matrix metalloproteinase 2 expression in human fibroblast-like synoviocytes by functional ionotropic glutamate receptors. Arthritis Rheum. 2007;56:2523-34.
101. Nam EJ, Sa KH, You DW, Cho JH, Seo JS, Han SW, et al. Up-regulated transforming growth factor beta-inducible gene h3 in rheumatoid arthritis mediates adhesion and migration of synoviocytes through alpha v beta3 integrin: Regulation by cytokines. Arthritis Rheum. 2006;54:2734-44.
102. Nowell MA, Richards PJ, Fielding CA, Ognjanovic S, Topley N, Williams AS, et al. Regulation of pre-B cell colony-enhancing factor by STAT-3-dependent interleukin-6 trans-signaling: implications in the pathogenesis of rheumatoid arthritis. Arthritis Rheum. 2006;54:2084-95.
103. Liu H, Huang Q, Shi B, Eksarko P, Temkin V, Pope RM. Regulation of Mcl-1 expression in rheumatoid arthritis synovial macrophages. Arthritis Rheum. 2006;54:3174-81.
104. Sweeney SE, Mo L, Firestein GS. Antiviral gene expression in rheumatoid arthritis: role of IKKepsilon and interferon regulatory factor 3. Arthritis Rheum. 2007;56:743-52.
105. Brown DA, Moore J, Johnen H, Smeets TJ, Bauskin AR, Kuffner T, et al. Serum macrophage inhibitory cytokine 1 in rheumatoid arthritis: a potential marker of erosive joint destruction. Arthritis Rheum. 2007;56:753-64.
106. Kelso EB, Ferrell WR, Lockhart JC, Elias-Jones I, Hembrough T, Dunning L, et al. Expression and proinflammatory role of proteinase-activated receptor 2 in rheumatoid synovium: ex vivo studies using a novel proteinase-activated receptor 2 antagonist. Arthritis Rheum. 2007;56:765-71.
107. Hayashi S, Miura Y, Nishiyama T, Mitani M, Tateishi K, Sakai Y, et al. Decoy receptor 3 expressed in rheumatoid synovial fibroblasts protects the cells against Fas-induced apoptosis. Arthritis Rheum. 2007;56:1067-75.
108. Maas K, Westfall M, Pietenpol J, Olsen NJ, Aune T. Reduced p53 in peripheral blood mononuclear cells from patients with rheumatoid arthritis is associated with loss of radiation-induced apoptosis. Arthritis Rheum. 2005;52:1047-57.
109. Juarranz Y, Gutiérrez-Cañas I, Santiago B, Carrión M, Pablos JL, Gomariz RP. Differential expression of vasoactive intestinal peptide and its functional receptors in human osteoarthritic and rheumatoid synovial fibroblasts. Arthritis Rheum. 2008;58:1086-95.
110. Bryl E, Vallejo AN, Matteson EL, Witkowski JM, Weyand CM, Goronzy JJ. Modulation of CD28 expression with anti-tumor necrosis factor alpha therapy in rheumatoid arthritis. Arthritis Rheum. 2005;52:2996-3003.
111. Hashimoto A, Tarner IH, Bohle RM, Gaumann A, Manetti M, Distler O, et al. Analysis of vascular gene expression in arthritic synovium by laser-mediated microdissection. Arthritis Rheum. 2007;56:1094-105.
112. Neidhart M, Jüngel A, Ospelt C, Michel BA, Gay RE, Gay S. Deficient expression of interleukin-10 receptor alpha chain in rheumatoid arthritis synovium: limitation of animal models of inflammation. Arthritis Rheum. 2005;52:3315-8.
113. Nakamachi Y, Kawano S, Takenokuchi M, Nishimura K, Sakai Y, Chin T, et al. MicroRNA-124a is a key regulator of proliferation and monocyte chemoattractant protein 1 secretion in fibroblast-like synoviocytes from patients with rheumatoid arthritis. Arthritis Rheum. 2009;60:1294-304.
114. Frosch M, Ahlmann M, Vogl T, Wittkowski H, Wulffraat N, Foell D, et al. The myeloid-related proteins 8 and 14 complex, a novel ligand of toll-like receptor 4, and interleukin-1beta form a positive feedback mechanism in systemic-onset juvenile idiopathic arthritis. Arthritis Rheum. 2009;60:883-91.
115. Manzo A, Vitolo B, Humby F, Caporali R, Jarrossay D, Dell'accio F, et al. Mature antigen-experienced T helper cells synthesize and secrete the B cell chemoattractant CXCL13 in the inflammatory environment of the rheumatoid joint. Arthritis Rheum. 2008;58:3377-87.
116. Vandooren B, Cantaert T, ter Borg M, Noordenbos T, Kuhlman R, Gerlag D, et al. Tumor necrosis factor alpha drives cadherin 11 expression in rheumatoid inflammation. Arthritis Rheum. 2008;58:3051-62.
117. Rioja I, Hughes FJ, Sharp CH, Warnock LC, Montgomery DS, Akil M, et al. Potential novel biomarkers of disease activity in rheumatoid arthritis patients: CXCL13, CCL23, transforming growth factor alpha, tumor necrosis factor receptor superfamily member 9, and macrophage colony-stimulating factor. Arthritis Rheum. 2008;58:2257-67.
118. Straub RH, Wolff C, Fassold A, Hofbauer R, Chover-Gonzalez A, Richards LJ, et al. Antiinflammatory role of endomorphins in osteoarthritis, rheumatoid arthritis, and adjuvant-induced polyarthritis. Arthritis Rheum. 2008;58:456-66.
119. Brentano F, Schorr O, Ospelt C, Stanczyk J, Gay RE, Gay S, et al. Pre-B cell colony-enhancing factor/visfatin, a new marker of inflammation in rheumatoid arthritis with proinflammatory and matrix-degrading activities. Arthritis Rheum. 2007;56:2829-39.
120. Grespan R, Fukada SY, Lemos HP, Vieira SM, Napimoga MH, Teixeira MM, et al. CXCR2-specific chemokines mediate leukotriene B4-dependent recruitment of neutrophils to inflamed joints in mice with antigen-induced arthritis. Arthritis Rheum. 2008;58:2030-40.
121. Geusens PP, Landewé RB, Garnero P, Chen D, Dunstan CR, Lems WF, et al. The ratio of circulating osteoprotegerin to RANKL in early rheumatoid arthritis predicts later joint destruction. Arthritis Rheum. 2006;54:1772-7.
122. Kang YM, Kim SY, Kang JH, Han SW, Nam EJ, Kyung HS, et al. LIGHT up-regulated on B lymphocytes and monocytes in rheumatoid arthritis mediates cellular adhesion and metalloproteinase production by synoviocytes. Arthritis Rheum. 2007;56:1106-17.
123. Matsunawa M, Isozaki T, Odai T, Yajima N, Takeuchi HT, Negishi M, et al. Increased serum levels of soluble fractalkine (CX3CL1) correlate with disease activity in rheumatoid vasculitis. Arthritis Rheum. 2006;54:3408-16.
124. van der Voort R, van Lieshout AW, Toonen LW, Slöetjes AW, van den Berg WB, Figdor CG, et al. Elevated CXCL16 expression by synovial macrophages recruits memory T cells into rheumatoid joints. Arthritis Rheum. 2005;52:1381-91.
125. Nanki T, Shimaoka T, Hayashida K, Taniguchi K, Yonehara S, Miyasaka N. Pathogenic role of the CXCL16-CXCR6 pathway in rheumatoid arthritis. Arthritis Rheum. 2005;52:3004-14.
126. Yao TC, Kuo ML, See LC, Ou LS, Lee WI, Chan CK, et al. RANTES and monocyte chemoattractant protein 1 as sensitive markers of disease activity in patients with juvenile rheumatoid arthritis: a six-year longitudinal study. Arthritis Rheum. 2006;54:2585-93
127. Edwards JR, Sun SG, Locklin R, Shipman CM, Adamopoulos IE, Athanasou NA, et al. A. LIGHT (TNFSF14), a novel mediator of bone resorption, is elevated in rheumatoid arthritis. Arthritis Rheum. 2006;54:1451-62.
128. Ikeuchi H, Kuroiwa T, Hiramatsu N, Kaneko Y, Hiromura K, Ueki K, et al. Expression of interleukin-22 in rheumatoid arthritis: potential role as a proinflammatory cytokine. Arthritis Rheum. 2005;52:1037-46
129. Weidler C, Struharova S, Schmidt M, Ugele B, Schölmerich J, Straub RH. Tumor necrosis factor inhibits conversion of dehydroepiandrosterone sulfate(DHEAS) to DHEA in rheumatoid arthritis synovial cells: a prerequisite for local androgen deficiency. Arthritis Rheum. 2005;52:1721-9.
130. Wijngaarden S, van de Winkel JG, Jacobs KM, Bijlsma JW, Lafeber FP, van Roon JA. A shift in the balance of inhibitory and activating Fcgamma receptors on monocytes toward the inhibitory Fcgamma receptor IIb is associated with prevention of monocyte activation in rheumatoid arthritis. Arthritis Rheum. 2004;50:3878-87.
131. Miranda-Carús ME, Benito-Miguel M, Balsa A, Cobo-Ibáñez T, Pérez de Ayala C, Pascual-Salcedo D, et al. Peripheral blood T lymphocytes from patients with early rheumatoid arthritis express RANKL and interleukin-15 on the cell surface and promote osteoclastogenesis in autologous monocytes. Arthritis Rheum. 2006 Apr;54(4):1151-64.
132. Kirkham BW, Lassere MN, Edmonds JP, Juhasz KM, Bird PA, Lee CS, Shnier R, Portek IJ. Synovial membrane cytokine expression is predictive of joint damage progression in rheumatoid arthritis: a two-year prospective study (the DAMAGE study cohort). Arthritis Rheum. 2006;54:1122-31.
133. Liao H, Wu J, Kuhn E, Chin W, Chang B, Jones MD, et al.Use of mass spectrometry to identify protein biomarkers of disease severity in the synovial fluid and serum of patients with rheumatoid arthritis. Arthritis Rheum. 2004;50:3792-803.
134. Sunahori K, Yamamura M, Yamana J, Takasugi K, Kawashima M, Makino H. Increased expression of receptor for advanced glycation end products by synovial tissue macrophages in rheumatoid arthritis. Arthritis Rheum. 2006;54:97-104.
135. Stanczyk J, Pedrioli DM, Brentano F, Sanchez-Pernaute O, Kolling C, Gay RE, et al. Altered expression of MicroRNA in synovial fibroblasts and synovial tissue in rheumatoid arthritis. Arthritis Rheum. 2008;58:1001-9.
136. Weidner S, Carl M, Riess R, Rupprecht HD. Histologic analysis of renal leukocyte infiltration in antineutrophil cytoplasmic antibody-associated vasculitis: importance of monocyte and neutrophil infiltration in tissue damage. Arthritis Rheum. 2004;50:3651-7.
137. Hu N, Westra J, Huitema MG, Bijl M, Brouwer E, Stegeman CA, et al. Coexpression of CD177 and membrane proteinase 3 on neutrophils in antineutrophil cytoplasmic autoantibody-associated systemic vasculitis: anti-proteinase 3-mediated neutrophil activation is independent of the role of CD177-expressing neutrophils. Arthritis Rheum. 2009;60:1548-57.
138. Abdulahad WH, Stegeman CA, Limburg PC, Kallenberg CG. Skewed distribution of Th17 lymphocytes in patients with Wegener's granulomatosis in remission. Arthritis Rheum. 2008;58:2196-205.
139. Casciola-Rosen L, Nagaraju K, Plotz P, Wang K, Levine S, Gabrielson E, et al. Enhanced autoantigen expression in regenerating muscle cells in idiopathic inflammatory myopathy. Exp Med. 2005;201:591-601.
140. López de Padilla CM, Vallejo AN, McNallan KT, Vehe R, Smith SA, Dietz AB, et al. Plasmacytoid dendritic cells in inflamed muscle of patients with juvenile dermatomyositis. Arthritis Rheum. 2007;56:1658-68.
141. Rouster-Stevens KA, Langman CB, Price HE, Seshadri R, Shore RM, Abbott K, et al. RANKL:osteoprotegerin ratio and bone mineral density in children with untreated juvenile dermatomyositis. Arthritis Rheum. 2007;56:977-83.
142. Elst EF, Klein M, de Jager W, Kamphuis S, Wedderburn LR, van der Zee R, et al. Hsp60 in inflamed muscle tissue is the target of regulatory autoreactive T cells in patients with juvenile dermatomyositis. Arthritis Rheum. 2008;58:547-55.
143. Richards TJ, Eggebeen A, Gibson K, Yousem S, Fuhrman C, Gochuico BR, et al. Characterization and peripheral blood biomarker assessment of anti-Jo-1antibody-positive interstitial lung disease. Arthritis Rheum. 2009;60:2183-92.
144. López De Padilla CM, Vallejo AN, Lacomis D, McNallan K, Reed AM. Extranodal lymphoid microstructures in inflamed muscle and disease severity of new-onset juvenile dermatomyositis. Arthritis Rheum. 2009;60:1160-72.
145. Grundtman C, Salomonsson S, Dorph C, Bruton J, Andersson U, Lundberg IE. Immunolocalization of interleukin-1 receptors in the sarcolemma and nuclei of skeletal muscle in patients with idiopathic inflammatory myopathies. Arthritis Rheum. 2007;56:674-87.
146. Fall N, Bove KE, Stringer K, Lovell DJ, Brunner HI, Weiss J, et al. Association between lack of angiogenic response in muscle tissue and high expression of angiostatic ELR-negative CXC chemokines in patients with juvenile dermatomyositis: possible link to vasculopathy. Arthritis Rheum. 2005;52:3175-80.
147. Waschbisch A, Wintterle S, Lochmüller H, Walter MC, Wischhusen J, Kieseier BC, et al. Human muscle cells express the costimulatory molecule B7-H3, which modulates muscle-immune interactions. Arthritis Rheum. 2008;58:3600-8.
148. Walsh RJ, Kong SW, Yao Y, Jallal B, Kiener PA, Pinkus JL, et al. Type I interferon-inducible gene expression in blood is present and reflects disease activity in dermatomyositis and polymyositis. Arthritis Rheum. 2007;56:3784-92.
149. Espinosa A, Zhou W, Ek M, Hedlund M, Brauner S, Popovic K, et al. The Sjogren's syndrome-associated autoantigen Ro52 is an E3 ligase that regulates proliferation and cell death. J Immunol. 2006;176:6277-85.
150. Sakai A, Sugawara Y, Kuroishi T, Sasano T, Sugawara S. Identification of IL-18 and Th17 cells in salivary glands of patients with Sjögren's syndrome, and amplification of IL-17-mediated secretion of inflammatory cytokines from salivary gland cells by IL-18. J Immunol. 2008 Aug 15;181(4):2898-906.
151. Barone F, Bombardieri M, Rosado MM, Morgan PR, Challacombe SJ, De Vita S, et al. CXCL13, CCL21, and CXCL12 expression in salivary glands of patients with Sjogren's syndrome and MALT lymphoma: association with reactive and malignant areas of lymphoid organization. J Immunol. 2008;180:5130-40.
152. Bombardieri M, Barone F, Humby F, Kelly S, McGurk M, Morgan P, et al. Activation-induced cytidine deaminase expression in follicular dendritic cell networks and interfollicular large B cells supports functionality of ectopic lymphoid neogenesis in autoimmune sialoadenitis and MALT lymphoma in Sjögren's syndrome. J Immunol. 2007;179:4929-38.
153. Gottenberg JE, Cagnard N, Lucchesi C, Letourneur F, Mistou S, Lazure T, et al. Activation of IFN pathways and plasmacytoid dendritic cell recruitment in target organs of primary Sjögren's syndrome. Proc Natl Acad Sci U S A. 2006;103:2770-5.
154. Nguyen CQ, Hu MH, Li Y, Stewart C, Peck AB. Salivary gland tissue expression of interleukin-23 and interleukin-17 in Sjögren's syndrome: findings in humans and mice. Arthritis Rheum. 2008;58:734-43.
155. Kwon YJ, Pérez P, Aguilera S, Molina C, Leyton L, Alliende C, et al. Involvement of specific laminins and nidogens in the active remodeling of the basal lamina of labial salivary glands from patients with Sjögren's syndrome. Arthritis Rheum. 2006;54:3465-75.
156. Pérez P, Kwon YJ, Alliende C, Leyton L, Aguilera S, Molina C, et al. Increased acinar damage of salivary glands of patients with Sjögren's syndrome is paralleled by simultaneous imbalance of matrix metalloproteinase 3/tissue inhibitor of metalloproteinases 1 and matrix metalloproteinase 9/tissue inhibitor of metalloproteinases 1 ratios. Arthritis Rheum. 2005;52:2751-60.
157. Hjelmervik TO, Petersen K, Jonassen I, Jonsson R, Bolstad AI. Gene expression profiling of minor salivary glands clearly distinguishes primary Sjögren's syndrome patients from healthy control subjects. Arthritis Rheum. 2005;52:1534-44.
158. Katsiougiannis S, Kapsogeorgou EK, Manoussakis MN, Skopouli FN. Salivary gland epithelial cells: a new source of the immunoregulatory hormone adiponectin. Arthritis Rheum. 2006;54:2295-9.
159. Wakamatsu E, Matsumoto I, Yasukochi T, Naito Y, Goto D, Mamura M, et al. Overexpression of phosphorylated STAT-1alpha in the labial salivary glands of patients with Sjögren's syndrome. Arthritis Rheum. 2006;54:3476-84.
160. Laine M, Porola P, Udby L, Kjeldsen L, Cowland JB, Borregaard N, et al. Low salivary dehydroepiandrosterone and androgen-regulated cysteine-rich secretory protein 3 levels in Sjögren's syndrome. Arthritis Rheum. 2007;56:2575-84.
161. Hu S, Zhou M, Jiang J, Wang J, Elashoff D, Gorr S, et al. Systems biology analysis of Sjögren's syndrome and mucosa-associated lymphoid tissue lymphoma in parotid glands. Arthritis Rheum. 2009;60:81-92.
162. Laine M, Virtanen I, Salo T, Konttinen YT. Segment-specific but pathologic laminin isoform profiles in human labial salivary glands of patients with Sjogren's syndrome. Arthritis Rheum. 2004;50:3968-73.
163. Hansen A, Reiter K, Ziprian T, Jacobi A, Hoffmann A, Gosemann M, et al. Dysregulation of chemokine receptor expression and function by B cells of patients with primary Sjögren's syndrome. Arthritis Rheum. 2005;52:2109-19.
164. Egerer T, Martinez-Gamboa L, Dankof A, Stuhlmüller B, Dörner T, Krenn V, et al. Tissue-specific up-regulation of the proteasome subunit beta5i (LMP7) in Sjögren's syndrome. Arthritis Rheum. 2006;54:1501-8.
165. Ping L, Ogawa N, Sugai S. Novel role of CD40 in Fas-dependent apoptosis of cultured salivary epithelial cells from patients with Sjögren's syndrome. Arthritis Rheum. 2005;52:573-81.
166. Båve U, Nordmark G, Lövgren T, Rönnelid J, Cajander S, Eloranta ML, et al. Activation of the type I interferon system in primary Sjögren's syndrome: a possible etiopathogenic mechanism. Arthritis Rheum. 2005;52:1185-95.
167. Manoussakis MN, Boiu S, Korkolopoulou P, Kapsogeorgou EK, Kavantzas N, Ziakas P, et al. Rates of infiltration by macrophages and dendritic cells and expression of interleukin-18 and interleukin-12 in the chronic inflammatory lesions of Sjögren's syndrome: correlation with certain features of immune hyperactivity and factors associated with high risk of lymphoma development. Arthritis Rheum. 2007;56:3977-88.
168. Ek M, Popovic K, Harris HE, Nauclér CS, Wahren-Herlenius M. Increased extracellular levels of the novel proinflammatory cytokine high mobility group box chromosomal protein 1 in minor salivary glands of patients with Sjögren's syndrome. Arthritis Rheum. 2006;54:2289-94.
169. Pers JO, d'Arbonneau F, Devauchelle-Pensec V, Saraux A, Pennec YL, Youinou P. Is periodontal disease mediated by salivary BAFF in Sjögren's syndrome? Arthritis Rheum. 2005;52:2411-4.
170. Daridon C, Pers JO, Devauchelle V, Martins-Carvalho C, Hutin P, Pennec YL, et al. Identification of transitional type II B cells in the salivary glands of patients with Sjögren's syndrome. Arthritis Rheum. 2006;54:2280-8.
